# Immunogenicity and safety of the first indigenously developed Indian tetravalent influenza vaccine (split virion) in healthy children (6 months to 17 years of age): a randomized, multicenter, phase III clinical trial

**DOI:** 10.1080/21645515.2020.1794683

**Published:** 2020-08-26

**Authors:** Sumantra Sarkar, Chandrakant Bokade, Kapil Garg, Ravi Kumar, Jayesh Sanmukhani, Ravindra Mittal

**Affiliations:** a Department of Pediatrics, IPGMER & SSKM Hospital, Kolkata, India; b Department of Pediatrics, Government Medical College and Hospital, Medical College Square, Nagpur, India; c Department of Paediatrics, Jay Kay Lon Hospital SMS Medical College, Jaipur, India; d Department of Pediatrics, Niloufer Hospital (Affiliated to Osmania Medical College), Hyderabad, India; e Department of Clinical Research and Regulatory Affairs, Cadila Healthcare Limited, India

**Keywords:** Tetravalent influenza vaccine, trivalent influenza vaccine, immunogenicity, cadila Healthcare Limited, vaxigrip, sanofi Pasteur, children, vaxi-flu 4

## Abstract

This phase III clinical trial was conducted to evaluate the immunogenicity and safety of the Tetravalent Influenza Vaccine (Split virion) I.P. (TetIV), containing two strains each of influenza A and B, developed indigenously in the country for the first time by M/s Cadila Healthcare Limited, India for use in the pediatric population (6 months −17 years of age), and compare it to that of a licensed seasonal Trivalent Influenza Vaccine (TriIV) of Sanofi Pasteur India Private Limited, containing two influenza A and one influenza B strains. Three hundred six subjects of either sex, 6 months to 17 years of age, were randomized in a 1:1 ratio to receive either TetIV or TriIV. Immunogenicity assessments (antibodies against A/H1N1, A/H3N2, B/Phuket, and B/Brisbane) were performed using the hemagglutination inhibition assay at baseline and 28 days after the last vaccination. TetIV was found to fulfill the criteria set by the United States Food and Drug Administration on the requirements of clinical data for licensure of seasonal inactivated influenza vaccines for the pediatric population. The seroconversion rates with TetIV were 94.6% for A/H1N1, 93.9% for A/H3N2, 91.2% for B/Brisbane, and 87.2% for B/Phuket strains. TetIV showed non-inferiority and superiority in immune response, as compared to TriIV, against the shared strains and an additional B strain, respectively. Both the vaccines were tolerated well by all the study participants, and an addition of the fourth strain in TetIV did not compromise the safety as compared to that of TriIV. The most common adverse event reported in both groups was fever.

## Introduction

Influenza is a highly infectious respiratory disease that affects individuals of all age groups, and the most vulnerable population include the young children, elderly, and those with chronic diseases. Children are at an increased risk of influenza infection compared to the general population, and influenza is associated with relatively high rates of serious illnesses in children of preschool age. In a recent modeling study estimating the global seasonal influenza-associated respiratory mortality conducted by using the WHO Global Health Estimates respiratory infection mortality rates, it has been estimated that a total of 291,243–645,832 seasonal influenza-associated respiratory deaths (4.0–8.8 per 100,000 individuals) occur globally in a year, while amongst these, 9243–105690 deaths occur among children younger than 5 years of age (2.1 to 23.8 per 100,000 population).^1^

According to published data from India, influenza contributes to around 5–10% of all acute respiratory infections. The reported incidence of influenza-associated upper respiratory tract infections was 100/1000 children per year and that of acute lower respiratory tract infections was only 4/1000 children per year.^2^ According to another Indian review, the influenza virus was responsible for approximately 1.5–14.5% of all acute respiratory infections.^3^ A community-based study from North India estimated the incidence of influenza episodes among children with acute respiratory infections to be around 147 per 1000 children per year and 155 per 1000 children per year, among children of 0–11 months and 12–23 months, respectively.^4^ Based on the epidemiology of influenza in Indian children and the increasing burden of the disease, especially in children less than 5 years of age, the Indian Academy of Pediatrics’ has recommended vaccinating all children between 6 months to 5 years of age with inactivated influenza vaccine once annually.^5^

Currently, two different formulations of influenza vaccines are approved and routinely used: a traditional Trivalent Influenza Vaccine (TriIV), which is composed of one A/H1N1 strain, one A/H3N2 strain, and one B strain; and a Tetravalent Influenza Vaccine (TetIV), which is composed of one A/H1N1 strain, one A/H3N2 strain, and influenza B strains from both the Victoria and Yamagata lineages. Various epidemiological reports from the past have shown that it is difficult to predict with acceptable accuracy which B lineage would be dominant in an upcoming season, and there have been frequent mismatches in the choice of B strain for vaccination, leading to an increased burden of disease despite vaccination with a TriIV.^6^ Based on these reports, it has been recommended by various scientific bodies, including the World Health Organization (WHO), to use TetIV, which has influenza B strains from both the Victoria and Yamagata lineages to improve protection against influenza B, thereby reducing the burden of seasonal influenza illnesses, hospitalization, and death.^7^

M/s Cadila Healthcare Limited is the first manufacturer to receive marketing authorization for a TetIV in India. The authorization was based on a Phase II/III clinical study conducted in adults and elderly population, the results of which have already been published in this journal earlier.^8^ This study was planned to evaluate the immunogenicity and safety of the inactivated influenza vaccine (split virion) I.P. (Tetravalent) of M/s Cadila Healthcare Limited in children aged 6 months to 17 years, and also compare those to that of an inactivated influenza vaccine (split Virion) I.P. (Trivalent) of M/s Sanofi Pasteur having the strains as recommended by the WHO for influenza vaccines for use in the 2017–2018 influenza season (northern hemisphere). To the best of our knowledge, this is the first clinical trial evaluating any tetravalent influenza vaccine (manufactured in India) in Indian children.

## Materials and methods

This prospective, randomized, single-blind, parallel-group, active-controlled, multicentre phase III clinical trial was conducted at four tertiary care centers in India from September 2017 to February 2018. The study was conducted by the pediatricians in compliance with the Indian Good Clinical Practice Guidelines and Ethical Principles of the Declaration of Helsinki. The study was approved by the Office of the Drug Controller General of India and was registered with the Clinical Trials Registry of India (www.ctri.nic.in; CTRI/2017/08/009204). The study was initiated after review and approval by the Institutional Ethics Committees at each of the four participating study centers. Written informed consent was obtained from either parent of each subject, and an additional written assent was acquired from subjects 7 years of age and older.

### Subjects

Subjects of either sex, 6 months to 17 years of age, were enrolled in the study. Parents of the subjects were required to have adequate literacy to complete the diary cards. Subjects were excluded from the study if they had any past history of hypersensitivity reaction, neurological disorder (Guillain–Barré syndrome or others), or any serious adverse event to any component of influenza vaccine, egg, chicken proteins, aminoglycoside antibiotics, history of administration of any influenza vaccine, or history of laboratory-confirmed influenza ever in the past. Other exclusion criteria were subjects with thrombocytopenia or any coagulation disorder, or on anticoagulation therapy; subjects with confirmed or suspected immunosuppressive or immunodeficiency disorder, or on any immunosuppressive or immunostimulant therapy; subjects with any clinically significant systemic disorder such as cardiovascular, respiratory, neurologic, gastrointestinal, hepatic, renal, endocrine, hematological, or immunological disorder; subjects with febrile illness (temperature ≥ 100.4°F) at the time of enrollment or acute respiratory pathology, or infections requiring systemic antibiotic or antiviral therapy during the preceding 7 days; subjects administered blood, blood-containing products, or immunoglobulins within the last 3 months or planned to be administered during the study period; subjects with history of any other vaccine administration within the last 30 days or planned to be administered during the study period; pregnant and lactating girls and female subjects not using acceptable contraceptive measures (double barrier methods, oral or injectable hormonal contraceptives, or surgical sterilization); and subjects who had participated in another clinical trial in the past 3 months. Subjects were permitted to use any medications for the treatment of concomitant diseases or adverse events during the study period that were not known to interact with the immunogenicity of the vaccine. However, a record of the same was maintained in the Case Record Form.

### Study procedures and vaccines

Subjects satisfying the eligibility criteria were randomized in a 1:1 ratio, as per the centralized computer-generated randomization schedule, to receive either TetIV or TriIV. As no TetIV was licensed for use in the pediatric population (<18 years of age) in India at the time of the study, a licensed TriIV was used for comparison. As the trial was conducted in the period 2017–2018, both the vaccines used in the study complied with the WHO recommendation for influenza vaccines for use in the 2017–2018 influenza season (Northern Hemisphere). TetIV contained A/Michigan/45/2015/(H1N1)-like virus, A/Hong Kong/4801/2014/H3N2-like virus, B/Brisbane/60/2008-like virus (B/Victoria), and B/Phuket/3073/2013-like virus (B/Yamagata); the TriIV contained A/Michigan/45/2015/(H1N1)-like virus; A/Hong Kong/4801/2014/H3N2-like virus, and B/Brisbane/60/2008-like virus (B/Victoria). Two different formulations of TetIV and TriIV were used in the study. The 0.5 mL formulation contained 15 µg of each antigen (virus strain) and was used in children aged ≥3 years whereas the 0.25 ml formulation contained 7.5 µg of each antigen (virus strain) and was used in children <3 years of age.

Subjects enrolled in the study were divided into three cohorts: cohort 1 (9–17 years), cohort 2 (3–8 years), and cohort 3 (6–35 months). The enrollment in cohorts was performed sequentially, wherein subjects in cohort 1 (9–7 years) were enrolled first and their 14-day safety data were reviewed by the Data Safety Monitoring Board (DSMB). Enrollment in cohorts 2 and cohort 3 was started after the recommendation of the DSMB. Subjects enrolled in cohort 1 (9–17 years) received a 0.5 ml single dose of either the TetIV or TriIV. Subjects enrolled in cohort 2 (3–8 years) received two doses (0.5 ml each, at least 28 days apart), whereas subjects in cohort 3 (6–35 months) received 2 doses (0.25 ml each, at least 28 days apart) of either vaccine. The dosing regimen was as per the recommendations of the Indian Academy of Pediatrics^5^ and the approved package insert of the other influenza vaccines in India and internationally.^9,10^ The vaccines were administered intramuscularly, injected into the deltoid muscle or the anterolateral aspect of the thigh, following aseptic precautions. Thereafter, subjects enrolled in cohort 1 were followed up for 42 days on an outpatient basis with scheduled visits on post-vaccination days 14, 28, and 42, whereas subjects enrolled in cohorts 2 and 3 were followed up for 70 days with scheduled visits on days 14, 28 (second vaccination), 42, 56, and 70.

A double-blind study was not planned because there was a difference in the formulation of the two study vaccines (TetIV in a vial and TriIV in a pre-filled syringe). All subjects were monitored for adverse events for at least 30 min following vaccination.

### Immunogenicity and safety assessments

Two milliliters of blood was collected from the subjects before vaccination and 28 days after last vaccination (i.e., 28 days for cohort 1 and 56 days for cohorts 2 and 3) for assessment of serum antibodies against the vaccine strains A/H1N1, A/H3N2, B/Victoria (B/Brisbane), and B/Yamagata (B/Phuket) by a validated hemagglutination inhibition assay. The assay utilized chicken red blood cells (RBCs) and a serum-virus incubation at room temperature (25°C) to provide optimal sensitivity and specificity for the vaccine antigens. Assays were performed at Cliantha Research Limited, India, by laboratory scientists who were blinded to vaccine assignment. Controls (including serum, RBC, and antigen controls) and participant sera were incubated with neuraminidase to eliminate nonspecific inhibitors. Spontaneous anti-species agglutinins were adsorbed by incubating the sera with a suspension of chicken RBCs. Ten twofold dilutions (starting at 1:10) of the treated sera were incubated with a previously titrated influenza virus solution at a concentration of 4 hemagglutination units/25 µL. Following incubation, the results of the assay were read, with the end point as the highest serum dilution in which complete inhibition of hemagglutination occurred. Both the pre-vaccination and post-vaccination sera of all the subjects were tested in the same assay and on the same plate. All serum samples were tested in duplicates.

The seroconversion rate was defined as the percentage of subjects with either a pre-vaccination HI titer <1:10 and a post-vaccination HI titer ≥1:40 or a pre-vaccination HI titer ≥1:10 and a minimum fourfold increase in post-vaccination HI antibody titer. The seroprotection rate was defined as the proportion of subjects with a post-vaccination titer ≥1:40. The geometric mean titer (GMT) of serum antibodies against the vaccine strains A/H1N1, A/H3N2, B/Victoria, and B/Yamagata were assessed at baseline (prior to vaccination) and at 28 days post-last vaccination. For the purpose of calculation, any antibody titer <10 (undetectable) was expressed as 5.

Diary cards were provided to the parents of the enrolled subjects to record solicited and unsolicited adverse events during the study period. The solicited events included pain, redness, swelling, fever, irritability, and drowsiness for children less than 3 years of age and pain, redness, swelling, fever, loss of appetite, headache, fatigue, muscle ache, joint pain, and shivering for children older than 3 years of age. Any other adverse event occurring during the follow-up (unsolicited) was also recorded on the diary card. Subjects enrolled in cohort 1 were given one diary card that was reviewed on each visit and collected back 42 days after vaccination (end of study visit). Subjects enrolled in cohorts 2 & 3 were given two diary cards, the first diary card was collected 28 days after the first vaccination, and a new card was given on the day of the second vaccination. This diary card was collected 42 days after the second vaccination, i.e., at the end of the study. The adverse events were graded from grades 1 to 3 based on the predefined criteria in the protocol.^11^

### Statistical analysis

The primary objective of this study was to demonstrate that the lower boundary of the 2-sided 95% confidence interval for the seroconversion and seroprotection rates for all the 4 strains, as calculated 28 days after the last vaccination in the TetIV group, exceeded 40% and 70%, respectively. This was adapted from the US FDA guidelines^10^ on the requirements of clinical data for licensure of seasonal inactivated influenza vaccines in the pediatric population, which is also routinely accepted by the Indian regulatory authorities for qualifying new influenza vaccines in India. The seroconversion and seroprotection rates for all four strains, as obtained with the test vaccine, were compared with those of the reference vaccine. The geometric mean titers for all four strains, 28 days after the last vaccination were also compared between the 2 groups. All data analyses were performed on the pooled data as the end point of vaccination in the three cohorts was the same as per the US FDA guidance, despite having different dosing regimens in the three age cohorts. This statistical approach was in line with the published literature.^12–15^

The seroconversion and seroprotection rates and GMTs in the two groups stratified by age (cohort 1: 9–17 years; cohort 2: 3–8 years, and cohort 3: 6–35 months) were also calculated.

Assuming the true seroconversion rate was at least 50% and the true seroprotection rate was at least 80% for each of the 4 strains, a sample size of 136 subjects in the test group provided a power of more than 99% to achieve the primary objective. Considering a drop-out rate of 10%, 306 subjects were enrolled in the 2 study groups. The seroprotection and seroconversion rates in the two groups were tested for non-inferiority for the three shared strains and superiority for the unshared B strain (B/Phuket of Yamagata lineage). The sample size of 306 subjects was also enough to achieve the secondary objective, i.e., to show non-inferiority of the 2 vaccines considering a one-sided alpha of 2.5%, non-inferiority margin of −10%, power of 80%, and a minimum seroconversion/seroprotection rate of 90% in both the groups.

To show non-inferiority/superiority, differences in the proportions (seroprotection rate/seroconversion rate) were calculated between the Test and Reference Groups; non-inferiority was considered when the lower bound of the 2-sided 95% CI of the difference between the proportions (Test Group – Reference Group) was not less than −10 percentage points, while superiority was considered when the lower bound of the 2-sided 95% CI of the difference between the proportions (Test Group – Reference Group) was more than 0 percentage points. To show non-inferiority/superiority in GMTs, the ratio of GMTs in the Test and Reference Groups was calculated; non-inferiority was considered when the lower bound of the 2-sided 95% CI of the ratio (Test Group/Reference Group) was not less than 0.67, and superiority was considered when the lower bound of the 2-sided 95% CI of the ratio (Test Group/Reference Group) was greater than 1. For subgroup analysis in various cohorts, standard statistical tests including Chi-square/Fischer’s exact test for proportions, and unpaired T-test for GMTs (on log_e_ converted data) were applied. A *P* value of less than 0.05 was considered significant.

Immunogenicity assessments were performed for both the Per Protocol (PP) Population (defined as all randomized subjects who had completed the trial with no violations, as per the protocol, with both pre- and post-vaccination immunological assessments) and the modified Intention to Treat (mITT) Population (defined as all the randomized subjects with both pre- and post-vaccination immunological assessments, including subjects with protocol violations). PP analysis was considered definitive and has been presented in the “Results” section. The safety population included all subjects who were administered the study vaccine and had been available for a 30 min observation period for safety assessment.

## Results

**Figure 1.**
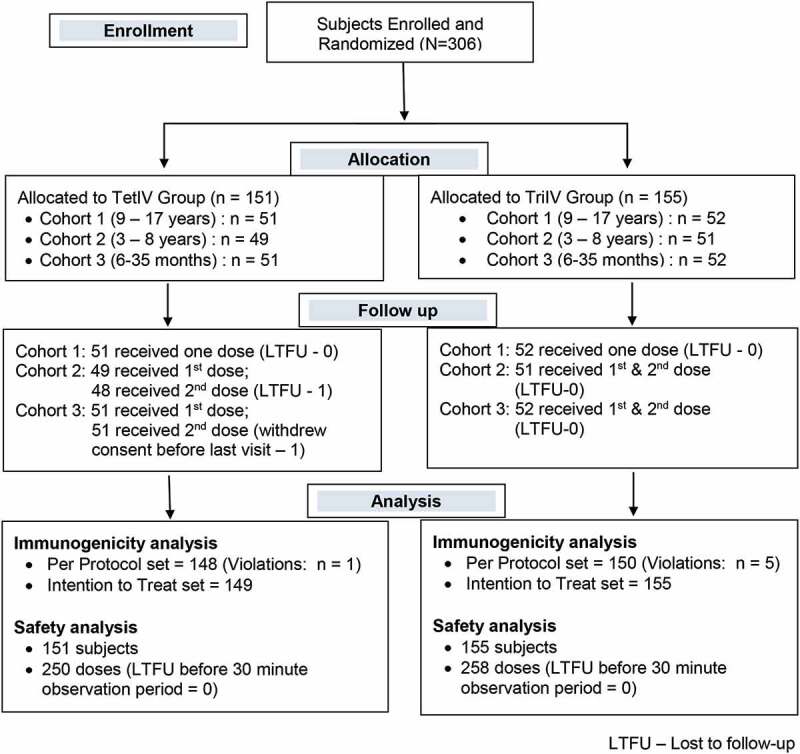
Flow of subjects in the study

Three hundred six subjects were enrolled in this randomized, single-blind, active-controlled, multicentre phase III clinical trial. One hundred fifty-one subjects were assigned to the TetIV group and 155 to the TriIV group. The 151 subjects in the TetIV group (cohort 1, 51; cohort 2, 49; and cohort 3, 51) received 250 doses of the vaccine (151 as the first dose and 99 as the second dose), whereas the 155 subjects in the TriIV group (cohort 1, 52; cohort 2, 51; and cohort 3, 52) received 258 doses of the vaccine (155 as the first dose and 103 as the second dose). All subjects completed the post-vaccination 30 min observation period after each dose and were thus considered for safety analysis. Among 151 subjects in the TetIV group, 3 subjects did not complete the study as per the protocol (one lost to follow up (LTFU), one withdrawal of consent, and one violation); hence, 148 subjects were considered for the per-protocol immunogenicity analysis (cohort 1, 50; cohort 2, 48; and cohort 3, 50). Among 155 subjects in the TriIV group, 5 subjects did not complete the study as per the protocol (5 violations); hence, 150 subjects were considered for the per-protocol immunogenicity analysis (cohort 1, 52; cohort 2, 49; cohort 3, 49). The flow of subjects through the study is shown in Figure 1. The demographic and baseline characteristics of the subjects are shown in Table 1.

**Table 1.** Demographic and baseline characteristics of all enrolled subjects

	TetIV Group	TriIV Group
**Total Study Population**		
	**N = 151**	**N = 155**
Age (Years)	6.0 ± 4.2	6.3 ± 4.6
Sex* Male	81 (53.6%)	89 (57.4%)
Female	70 (46.4%)	66 (42.6%)
Height (cm)	110.6 ± 29.8	110.5 ± 29.8
Weight (kg)	21.6 ± 14.4	21.7 ± 14.0
**Cohort 1 (9 to 17 years)**		
	**N = 51**	**N = 52**
Age (Years)	11.3 ± 1.9	12.0 ± 2.4
Sex* Male	32 (62.8%)	35 (67.3%)
Female	19 (37.3%)	17 (32.7%)
Height (cm)	144.9 ± 12.2	145.2 ± 12.9
Weight (kg)	38.7 ± 12.0	37.0 ± 13.5
**Cohort 2 (3 to 8 years)**		
	**N = 49**	**N = 51**
Age (Years)	4.8 ± 1.2	5.1 ± 1.5
Sex* Male	27 (55.1%)	27 (52.9%)
Female	22 (44.9%)	24 (47.1%)
Height (cm)	106.8 ± 16.5	106.5 ± 12.9
Weight (kg)	15.7 ± 3.1	17.2 ± 4.5
**Cohort 3 (6 to 35 months)**		
	**N = 51**	**N = 52**
Age (Months)	21.1 ± 8.7	20.1 ± 8.6
Sex* Male	22 (43.1%)	27 (51.9%)
Female	29 (56.9%)	25 (48.1%)
Height (cm)	80.0 ± 9.1	79.7 ± 11.6
Weight (kg)	10.3 ± 2.3	10.6 ± 2.6

Data expressed as mean ± SD

*Data expressed as n (%)

### Immunogenicity

Table 2 presents the primary immunogenicity end point data, i.e., the seroconversion and seroprotection rates of all four strains at 28 days after the last vaccination in the TetIV group. The lower bound of the 95% CI of the seroconversion and seroprotection rates was greater than the recommended limit of 40% and 70%, respectively, for all 4 strains in the TetIV group, and thus fulfilling the primary criteria as per the US FDA guidance^16^ on the requirements of clinical data for licensure of seasonal inactivated influenza vaccines in pediatric populations.

**Table 2.** Seroconversion and seroprotection rates, and GMTs in the two groups, 28 days after the last vaccination

Immunogenicity end point	TetIV Group (N = 148)	TriIV Group (N = 150)	TetIV – TriIV or TetIV/TriIV^^^
**A/Michigan (A/H1N1)**			
Seroconversion*	140 (94.6%) (89.6% – 97.6%)	141 (94.0%) (88.9% – 97.2%)	0.6% (−4.7% – 5.9%)
Seroprotection*	148 (100.0%) (97.5% – 100.0%)	150 (100.0%) (97.6% – 100.0%)	0.0% (0.0% – 0.0%)
GMT**^#^**	935.3 (797.9–1096.3)	1030.1 (902.0–1176.5)	0.91 (0.73–1.12)
**A/Hong Kong (A/H3N2)**			
Seroconversion*	139 (93.9%) (88.8% – 97.2%)	141 (94.0%) (88.9% – 97.2%)	−0.1% (−5.3% – 5.5%)
Seroprotection*	147 (99.3%) (96.3% – 100.0%)	150 (100.0%) (97.6% – 100.0%)	−0.7% (−2.0% – 0.6%)
GMT**^#^**	1543.7 (1264.1–1885.3)	1689.0 (1437.4–1984.6)	0.91 (0.71–1.18)
**B/Brisbane (B/Victoria)**			
Seroconversion*	135 (91.2%) (85.4% – 95.2%)	125 (83.3%) (76.4% – 88.9%)	7.9% (0.3% – 15.5%)
Seroprotection*	138 (93.2%) (87.9% – 96.7%)	127 (84.7%) (77.9% – 90.0%)	8.5% (1.5% – 15.7%)
GMT**^#^**	203.2 (162.5–254.1)	201.6 (156.7–259.3)	1.01 (0.72–1.41)
**B/Phuket (B/Yamagata)**			
Seroconversion*	129 (87.2%) (80.7% – 92.1%)	51 (34.0%) (26.5% – 42.2%)	53.2% (42.1% – 64.3%)
Seroprotection*	131 (88.5%) (82.2% – 93.2%)	58 (38.7%) (30.8% – 47.0%)	49.8% (38.9% – 60.8%)
GMT**^#^**	91.2 (76.0–109.5)	22.6 (18.4–27.7%)	4.04 (3.08–5.31)

*Data presented as n (%) (95% CI)

^#^Data presented as geometric mean (95% CI)

^TetIV – TriIV values given for seroconversion and seroprotection rates; TetIV/TriIV ratio given for GMT 28 days after the last dose

A comparison of the seroconversion rate, seroprotection rate, and GMTs in the two groups is also shown in Table 2. The seroconversion and seroprotection rates at 28 days after the last dose of vaccine for the 3 shared strains in the 2 vaccines, i.e., A/Michigan (A/H1N1), A/Hong Kong (A/H3N2), and B/Brisbane (B/Victoria) met the non-inferiority criteria, i.e., the lower limit of 95% CI of the difference in the seroconversion/seroprotection rate was more than −10%. The seroconversion and seroprotection rates of the B/Brisbane strain of TetIV even reached superior levels. GMT at 28 days after the last vaccination for the three common strains also met the non-inferiority criteria, i.e., the lower limit of 95% CI of the GMT ratio was more than 0.67. The TetIV was superior to TriIV for all the three immunogenicity parameters, i.e., the seroconversion and seroprotection rates, and GMT at 28 days after the last dose of vaccination for the B/Phuket (Yamagata lineage) strain. This was because of the presence of additional B/Phuket strain in TetIV as compared to TriIV.

Table 3, 4, and 5 present the age-stratified immunogenicity data in the three cohorts of the study. The results of all three immunogenicity parameters (seroconversion rate, seroprotection rate, and the GMT 28 days after the last dose of vaccination) for the two A strains (A/H1N1 and A/H3N2) were similar in the three cohorts, as in the pooled data. However, seroconversion and seroprotection rates, and GMT 28 days after the last dose of vaccination were significantly better with the B/Brisbane (B/Victoria) strain in TetIV in cohort 3 subjects, whereas there was no significant difference among the subjects in cohorts 1 and 2 (the significant difference among cohort 3 subjects may have skewed the seroconversion and seroprotection rates toward superiority in the pooled data). This could be attributed to the batch-to-batch variation in the antigenic content in the vaccine formulations (0.25 mL formulation was used for cohort 3 subjects whereas 0.5 mL formulation was used for the subjects in cohorts 1 and 2). TetIV was superior to TriIV in terms of all three immunogenicity parameters for the B/Phuket (Yamagata lineage) strain in all three cohorts, as was in the pooled data.

**Table 3.** Seroconversion rate 28 days after the last vaccination in the three cohorts

	TetIV Group	TriIV Group	*P* value
**A/Michigan (A/H1N1)**			
Cohort 1	45/50 (90.0%) (78.2% – 96.7%)	48/52 (92.3%) (81.5% – 97.9%)	0.95
Cohort 2	45/48 (93.8%) (82.8% – 98.7%)	45/49 (91.8%) (80.4% – 97.7%)	0.98
Cohort 3	50/50 (100.0%) (92.9% – 100.0%)	48/49 (98.0%) (89.1% – 99.9%)	0.99
**A/Hong Kong (A/H3N2)**			
Cohort 1	47/50 (94.0%) (83.5% – 98.7%)	45/52 (86.5%) (74.2% – 94.4%)	0.35
Cohort 2	43/48 (89.6%) (77.3% – 96.5%)	47/49 (95.9%) (86.0% – 99.5%)	0.42
Cohort 3	49/50 (98.0%) (89.4% – 99.9%)	49/49 (100.0%) (92.7% – 100.0%)	0.99
**B/Brisbane (B/Victoria)**			
Cohort 1	44/50 (88.0%) (75.7% – 95.5%)	46/52 (88.5%) (76.6% – 95.6%)	0.81
Cohort 2	43/48 (89.6%) (77.3% – 96.5%)	43/49 (87.8%) (75.2% – 95.4%)	0.97
Cohort 3	48/50 (96.0%) (86.3% – 99.5%)	36/49 (73.5%) (58.9% – 85.1%)	0.004
**B/Phuket (B/Yamagata)**			
Cohort 1	44/50 (88.0%) (75.7% – 95.5%)	20/52 (38.5%) (25.3% – 53.0%)	<0.01
Cohort 2	40/48 (83.3%) (69.8% – 92.5%)	22/49 (44.9%) (30.7% – 59.8%)	0.0002
Cohort 3	45/50 (90.0%) (78.2% – 96.7%)	9/49 (18.4%) (8.8% – 32.0%)	<0.0001

Data presented as n/N (%) (95% CI)

*P* value based on chi-square test

**Table 4.** Seroprotection rate 28 days after the last vaccination in the three cohorts

	TetIV Group	TriIV Group	*P* value
**A/Michigan (A/H1N1)**			
Cohort 1	50/50 (100.0%) (92.9% – 100.0%)	52/52 (100.0%) (93.2% – 100.0%)	NA
Cohort 2	48/48 (100.0%) (92.6% – 100.0%)	49/49 (100.0%) (92.7% – 100.0%)	NA
Cohort 3	50/50 (100.0%) (92.9% – 100.0%)	49/49 (100.0%) (92.7% – 100.0%)	NA
**A/Hong Kong (A/H3N2)**			
Cohort 1	50/50 (100.0%) (92.9% – 100.0%)	52/52 (100.0%) (93.2% – 100.0%)	NA
Cohort 2	48/48 (100.0%) (92.6% – 100.0%)	49/49 (100.0%) (92.7% – 100.0%)	NA
Cohort 3	49/50 (98.0%) (89.4% – 99.9%)	49/49 (100.0%) (92.7% – 100.0%)	0.99
**B/Brisbane (B/Victoria)**			
Cohort 1	46/50 (92.0%) (80.8% – 97.8%)	47/52 (90.4%) (79.0% – 96.8%)	0.95
Cohort 2	44/48 (91.7%) (80.0% – 97.7%)	44/49 (89.8%) (77.8% – 96.6%)	0.97
Cohort 3	48/50 (96.0%) (86.3% – 99.5%)	36/49 (73.5%) (58.9% – 85.1%)	0.004
**B/Phuket (B/Yamagata)**			
Cohort 1	44/50 (88.0%) (75.7% – 95.5%)	23/52 (44.2%) (30.5% – 58.7%)	<0.01
Cohort 2	42/48 (87.5%) (74.8% – 95.3%)	25/49 (51.0%) (36.3% – 65.6%)	0.0002
Cohort 3	45/50 (90.0%) (78.2% – 96.7%)	10/49 (20.4%) (10.2% – 34.3%)	<0.0001

Data presented as n/N (%) (95% CI)

*P* value based on chi-square test

**Table 5.** Geometric Mean Titer 28 days after the last vaccination in the three cohorts

	TetIV Group	TriIV Group	*P* value
**A/Michigan (A/H1N1)**			
Cohort 1	1245.0 (1016.5–1524.9)	1368.2 (1129.1–1657.9)	0.50
Cohort 2	854.3 (631.8–1155.2)	964.6 (778.2–1195.6)	0.51
Cohort 3	766.4 (561.9–1045.3%)	814.0 (620.6–1067.6)	0.77
**A/Hong Kong (A/H3N2)**			
Cohort 1	2137.8 (1760.5–2596.0)	1884.0 (1536.8–2309.7)	0.37
Cohort 2	2031.9 (1456.4–2834.8	2286.1 (1760.4–2968.8)	0.58
Cohort 3	856.3 (557.7–1314.7)	1111.2 (789.0–1564.9)	0.34
**B/Brisbane (B/Victoria)**			
Cohort 1	232.6 (145.2–372.7)	307.5 (201.5–469.3)	0.38
Cohort 2	169.5 (117.8–243.9)	227.9 (154.6–335.9)	0.27
Cohort 3	211.1 (151.3–294.5)	113.9 (70.8–183.4)	0.04
**B/Phuket (B/Yamagata)**			
Cohort 1	117.9 (80.7–172.5)	27.9 (19.3–40.4)	<0.0001
Cohort 2	88.5 (64.7–121.9)	30.1 (20.7–43.8)	<0.0001
Cohort 3	72.6 (56.6–93.1%)	13.5 (10.1–17.9)	<0.0001

Data Expressed as GMT (95% CI)

*P* value based on Unpaired T-Test

### Safety

**Figure 2.**
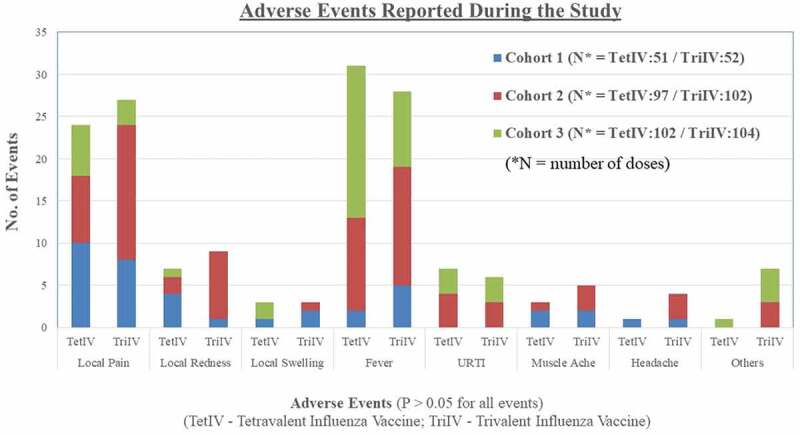
Adverse events reported post influenza vaccination

Seventy-seven adverse events were reported in 40 subjects in the TetIV group, and 89 adverse events were reported in 41 subjects in the TriIV group. The most common local adverse event reported during the study was pain at the site of injection, and the most common systemic adverse event reported during the study was fever in both groups. The details of the adverse events reported during the study are shown in Figure 2. There was no significant difference in the profile of adverse events between the two groups. Sixty-three of 77 adverse events in the TetIV group and 80 of 89 adverse events reported in the TriIV group were “mild” in severity. Most of the adverse events lasted for 1–3 days (85.7% in the TetIV group and 82.0% in the TriIV group). All adverse events resolved completely, with or without symptomatic treatment, during the study period. No “serious” or “severe” adverse events were reported in the subjects during the study.

Subgroup analysis of safety data showed that there was no difference in the adverse events reported between groups in the three cohorts during the study. Pain was more commonly reported in cohort 1, whereas fever was more commonly reported in subjects of cohorts 2 and 3 (proportion of events). The adverse events occurring after the first and second doses of the vaccine were also analyzed separately. Although not statistically significant, the proportion of subjects with pain and fever was lower after the second dose of the vaccine as compared to that after the first dose of the vaccine (data not shown).

## Discussion

This study presents the results of the first comparative phase III clinical trial conducted to assess the immunogenicity and safety of TetIV of M/s Cadila Healthcare Limited, the only TetIV that has been developed indigenously in the country, for the Indian pediatric population. The study showed that the vaccine was able to elicit a strong hemagglutination inhibition (HI) antibody response against all four strains, i.e., A/H1N1, A/H3/N2, B/Victoria (B/Brisbane), and B/Yamagata (B/Phuket), and fulfilled the requirements of clinical data laid by the US FDA for licensure of seasonal inactivated influenza vaccines in the pediatric population. TetIV had a superior antibody response in the pediatric population for the additional B strain (B/Yamagata), compared to the TriIV in which this strain was absent. In our previous publication reporting the immunogenicity and safety results of a Phase II/III clinical study conducted in adults and elderly populations with the same TetIV,^8^ we showed that the addition of the new antigen (additional B strain) did not interfere with the immune response of the existing vaccine antigens and did not change the safety profile of the vaccine. Similar results have been reported with the TetIV of Cadila Healthcare Limited in the pediatric population. The non-inferiority and superiority of TetIV as compared to the TriIV against the shared strains and an additional B strain, respectively, support the use of the TetIV for vaccination against seasonal influenza in children aged 6 months and above, as a strategy to potentially improve protection against influenza B.

Based on the burden of influenza B and inability to accurately predict which influenza B lineage would circulate in the subsequent season, there is a clear need to move from a trivalent to a tetravalent influenza vaccine that has both influenza B lineages as this could reduce the incidence of seasonal influenza.^6^ Influenza B is associated with substantial morbidity and hospitalization and is reported to be a disproportionate cause of influenza deaths, especially in children. During the 2010–2011 season in the US, although influenza B accounted for only 26% of infections, 44 of 115 (38%) influenza-related deaths in children were associated with influenza B.^13^ In a review of literature of the epidemiology of influenza disease in 15 countries in the Asia-Pacific region, influenza B was identified and associated with between 0% and 92% of laboratory‐confirmed influenza cases in any one season. Influenza type B was associated with more illness in children aged 1–10 years as compared to the other age groups. Although the data on the two different lineages of B strain are limited in the Asian countries, it has been found that both the B lineages co-circulate in countries where surveillance data are available. Also, a mismatch between the circulating B strain and vaccine strain has also been observed in all countries where the data is available.^17^ Similar epidemiological data on co-circulation of both the lineages of influenza B strain in a single season have also been reported in India,^18,19^ further emphasized the need to replace TriIV with the TetIV for routine immunization of children. Moreover, in seasons in which influenza B circulation is minimal or B viruses are well matched to the trivalent vaccine strain, vaccination with a TetIV would still be beneficial to the individual by increasing immunity to both lineages of influenza B, with potential clinical benefit in subsequent seasons. This accumulated immunity is more relevant for influenza B than for influenza A because antigenic drift is more limited with influenza B viruses.^20^

The results of this study have shown that the TetIV vaccine of Cadila Healthcare Limited provides robust immunity against all four strains in the vaccine, and the data are comparable to those of internationally published trials with TetIV in children and infants. In one randomized controlled trial to evaluate the immunogenicity and safety of an inactivated quadrivalent influenza vaccine candidate of GlaxoSmithKline vaccines conducted in Canada, the United States, Mexico, Spain, and Taiwan, the seroprotection rates were 96.8% for A/California/7/2009 (H1N1), 92.9% for A/Victoria/210/2009 (H3N2), 95.4% for B/Brisbane/60/2008 (Victoria), and 99.0% for B/Florida/4/2006 (Yamagata), in children 3–17 years of age, whereas the corresponding seroprotection rates in children 6 to 35 months of age were 89.6%, 74.5%, 88.0%, and 96.5%, respectively.^12^ In another similar study to evaluate the immunogenicity and safety of an inactivated quadrivalent influenza vaccine candidate of GlaxoSmithKline Vaccines conducted in the Czech Republic, France, Germany, the Philippines, and the US, the seroprotection rates were 96.6% for A/California/7/2009 (H1N1), 98.0% for A/Victoria/210/2009 (H3N2), 97.3% for B/Brisbane/60/2008 (Victoria), and 99.2% for B/Brisbane/3/2007 (Yamagata) in children aged 3–17 years, whereas the corresponding seroprotection rates in children 6 to 35 months of age were 79.9%, 72.2%, 71.4%, and 90.6%, respectively.^13^ Both published studies have shown that influenza vaccines are only moderately immunogenic in children <3 years of age who have had limited previous exposure to vaccines and viruses. However, the immunogenicity results with the TetIV vaccine of Cadila Healthcare Limited in cohort 3, i.e., children between 6 and 35 months of age have also been very robust and similar to the immunogenicity results of subjects in cohorts 1 and 2.

The results of our study also highlight the importance of vaccination in young children, especially those aged 6–35 months. As can be seen from the results with the additional B strain (B/Phuket) in the subgroup analysis, the seroconversion and seroprotection rates in the TriIV group ranged from 40–50% in cohorts 1 and 2, corresponding to only 20% in cohort 3. This higher seroconversion and seroprotection rates in cohorts 1 and 2 even for the strain not present in the TriIV, can be attributed to environmental exposure, subclinical infection (the study was conducted in peak season for influenza in India, i.e., September to February), and partly to cross-reactivity. As can be noted from the poor seroconversion/seroprotection rates, an immune response is not elicited in infants aged 6–35 months, probably because of the immature immune system not responding to the environmental exposure and subclinical infection. However, vaccination in this age group elicits a good immune response resulting in seroconversion and seroprotection rates similar to those in cohorts 1 and 2 after vaccination, thereby further highlighting the importance of influenza vaccination, especially in this age group.

The inclusion of an additional antigen in TetIV could have led to higher reactogenicity and adverse events; however, the adverse event profiles of both TetIV and TriIV were found to be similar in this study. Moreover, there was no significant difference in the adverse event profiles among the three cohorts. The incidence of pain was found to be higher in cohorts 1 and 2 than that in cohort 3, which was expected as children in cohorts 2 and 3 are more likely to share and complain about pain as compared to children in the lower age group. The incidence of fever was more common in younger children aged 6–35 months as compared to children in the higher age group. These results suggest that the addition of the fourth strain in TetIV did not compromise the safety as compared to TriIV. The safety profile of TetIV of M/s Cadila Healthcare Limited was similar to that of the other approved and marketed tetravalent/quadrivalent influenza vaccines internationally.^9,10^

This is the first indigenously developed tetravalent influenza vaccine to have received marketing authorization for use in children in the country. Until now, all inactivated influenza vaccines that were marketed in India were imported from other countries. Local manufacturing of the vaccine will help reduce cost and increase the usage, thereby promoting public health in the region and ensuring preparedness in case of a pandemic.

One of the limitations of the study is that it was only a serological study and further efficacy studies would be required to evaluate the impact of including both the lineages of influenza type B strain in reducing the burden of influenza disease in the community. Another limitation of the study was its single-blind design, which could have led to some bias in the reporting of adverse events. A double-blind study could not be conducted as there was a difference in the packaging of the two vaccines (TetIV in a vial and TriIV in a pre-filled syringe) and a double-dummy design would have led to the administration of two injections in all the participants, which could have raised ethical concerns.

## Conclusion

The results of this randomized, single-blind, multicentre, phase III clinical trial in the pediatric population showed that the Inactivated Tetravalent Influenza Vaccine (split virion) I.P. of M/s Cadila Healthcare Limited provided non-inferior immunogenicity against the shared strains, and superior immunogenicity against the additional B strain compared to that of the marketed Inactivated Trivalent Influenza Vaccine of M/s Sanofi Pasteur India Private Limited, without affecting the safety profile of the vaccine. These results show that TetIV could prevent influenza B lineage mismatch and potentially improve protection against influenza B in children.
